# Experimental Evaluation of Ethanolic Extract of *Carapa guianensis* L. Leaf for Its Wound Healing Activity Using Three Wound Models

**DOI:** 10.1093/ecam/nep160

**Published:** 2011-03-17

**Authors:** B. Shivananda Nayak, Joel Kanhai, David Malcolm Milne, Lexley Pinto Pereira, William H. Swanston

**Affiliations:** ^1^Department of Preclinical Sciences, The University of West Indies, Faculty of Medical Sciences, Biochemistry Unit, EWMSC, Trinidad and Tobago; ^2^Department of Para Clinical Sciences, Faculty of Medical Sciences, The University of the West Indies, St. Augustine, Trinidad and Tobago; ^3^Diagnostic Laboratory Services, North Central Regional Health Authority, Trinidad and Tobago

## Abstract

The leaves of *Carapa guianensis* have been used to treat ulcers, skin parasites, and skin problems. The ethanolic extract of *C. guianensis* leaf was evaluated for its antibacterial and wound healing activity using excision, incision and dead space wound models in rats. The animals were randomly divided into two groups (*n* = 6) in all the models. In the excision wound model test group animals were treated topically with the leaf extract (250 mg kg^−1^ body weight) whereas, control animals were treated with petroleum jelly. In the incision and dead space wound models, the test group animals were treated with extract (250 mg kg^−1^ day^−1^) orally by mixing in drinking water and the control group animals were maintained with plain drinking water. Healing was assessed by the rate of wound contraction, period of epithelialization, skin breaking strength, granulation tissue weight and hydoxyproline content. On Day 15 extract-treated animals exhibited 100% reduction in the wound area when compared to controls (95%) with significant decrease in the epithelialization period. The extract failed to demonstrate antibacterial activity. Skin breaking strength (*P* < .001), wet (*P* < .002) and dry (*P* < .02) granulation tissue and hydroxyproline content (*P* < .03) were significantly higher in extract treated animals. The increased rate of wound contraction, skin breaking strength and hydroxyproline content supports potential application of *C. guianensis* in wound healing.

## 1. Introduction

Traditional herbal medicine practitioners have described the healing properties of various wild plants [1, 2]. Various healing constituents in these plants have prompted researchers to examine them with a view to determine their potential wound healing activities.

Healing of skin wounds is a complex process which recruits the collaborative efforts of different tissues of varying cell lineage. The behavior of each of the contributing cell types during the phases of proliferation, migration, matrix synthesis and contraction, as well as the growth factor and matrix signals present at a wound site are now well understood [3]. Following an injury, a series of events takes place in a predictable fashion to repair the damage. In the subsequent inflammatory response following an injury the cells below the dermis (the deepest skin layer) begin to increase collagen (connective tissue) production reaching the last stage of regeneration of, epithelial tissue (the outer skin layer) [4].

*Carapa guianensis* L. (*Meliaceae*) is also known as Andiroba. The leaves have been used for fever and the tea made from this plant is applied externally for ulcers, skin parasites and other skin problems. Traditional forest dwellers particularly those dwelling on the river bank in Brazil called *caboclos*, make a medicinal soap using crude Andiroba oil, wood ash and cocoa skin residue. This soap is especially recommended for the treatment of skin diseases. Andiroba oil is also applied directly on joints to relieve arthritic pain, and mixed with hot water and human milk it is used as drops for ear infections. Many of these uses continue today in the Brazilian herbal medicine systems either in pure form or mixed with other oils or natural products. Brazilians apply andiroba oil externally to wounds and bruises, use it as a massage oil and natural insect repellant, and employ it topically for many skin diseases and conditions, including psoriasis.

All parts of the *C. guianensis* tree have a bitter taste attributed to a group of terpene chemicals called *meliacins*, which are very similar to the bitter antimalarial chemicals found in tropical plants. One of these meliacins, named *gedunin*, has recently been documented to have antiparasitic properties and an antimalarial effect equal to that of quinine. Chemical analysis of *C. guianensis* oil, leaves has also identified the presence of another group of chemicals called limonoids. The anti-inflammatory and insect repellent properties of andiroba oil are attributed to the presence of these limonoids [5], including a novel one which has been named *andirobin*. Another limonoid called *epoxyazadiradione* is found in *C. guianensis* oil. The three chemicals present in Andiroba have been found to have antiparasitic and insecticidal actions [6, 7]. *Carapa guianensis* oil is well known in Brazil and widely employed to heal many skin conditions and as a natural insect repellant. North American practitioners and consumers are just beginning to learn about the powerful healing properties of *C. guianensis*. Andiroba oil can be applied topically several times daily to rashes, muscle/joint aches and injuries, wounds [8] insect bites, boils and ulcers.

However, there is not enough scientifically proven data to support the wound healing and antimicrobial activities of *C. guianensis* in literature. We undertook the present study to explore the antimicrobial and wound healing effects of *C*. *guianensis* leaf extract.

## 2. Methods

### 2.1. Plant Material and Extract Preparation

The *C. guianensis* leaf (625 g) was collected locally in March 2008 and identified by the plant taxonomist and curator, National Herbarium of Trinidad and Tobago, The University of the West Indies, St. Augustine, Trinidad and a voucher specimen was deposited at the herbarium (specimen number: TRIN 36521). The leaves were washed with tap water and finally with deionized water. After shade drying they were ground into a powder using an electric blender. The fine powder (620 g) was suspended in 4000 ml of ethanol for 48 h at room temperature. The mixture was filtered using a fine cloth and the filtrate was placed in a water bath to dry at 40°C. The dried residue (60 g) was used for the study [9]. The extract was subjected to preliminary phytochemical and microbial tests.

### 2.2. Phytochemical Screening Methods

#### 2.2.1. Saponins

The extract (2 g) was boiled with 20 ml water for 4 min; the mixture was cooled and mixed vigorously and left for few minutes. The formation of frothing indicates the presence of saponins [10].

*Test for tannins*: To an aliquot of the extract (dissolved in water) 2 ml of ferric chloride (1%) was added. Color development from red brown to blue black indicates the presence of tannins [10].

#### 2.2.2. Triterpenes

The extract (1 g) was mixed with 10 ml chloroform and warmed at 55°C for 30 min. Few drops (1-2 ml) of concentrated sulfuric acid were added and mixed well. The appearance of a reddish brown color indicates the presence of triterpenes [11].

#### 2.2.3. Test for Sterols

The extract (1 g) was mixed with 10 ml chloroform and warmed at 55°C for 30 min. Few drops (1–2 ml) of concentrated sulfuric acid were added and mixed well. The appearance of reddish brown color indicates the presence of sterols [11].

#### 2.2.4. Alkaloids

The extract (1 g) was boiled with 50 ml methanol for 20 min in a water bath and the cooled filtrate was tested separately with Mayer's, Wagner's Hager's and ammonium reineckate reagents. Cloudy precipitate of the alcoholic layer indicates the presence of alkaloids [11].

#### 2.2.5. Flavonoids

About 1 g of extract was boiled with 10 ml ethyl acetate over a steam bath for 3 min. The filtrate of about 4 ml was mixed with 1 ml of dilute ammonia solution and a yellow precipitate indicates the presence of flavonoids [10].

Thin layer chromatography of the aqueous extract on silica gel was done using the medium chloroform: methanol (9 : 1 v/v) and chloroform: acetone (1 : 1 v/v) as the mobile phase.

### 2.3. Antimicrobial Activity

*Pseudomonas aeruginosa* (ATCC 27853), *Klebsiella pneumonia* (ATCC 700603), *Enterococcus fecalis* (ATCC 29212), *Escherichia coli* (ATCC 25922), *Staphylococcus aureus* (ATCC25923) and Methicilin-resistant *S. aureus* (ATCC 43300) were tested for antibacterial sensitivity. The bacterial strains were obtained from fresh colonies grown on MacConkey and blood agar plates. The sensitivity testing was done using Muller Hinton Agar plates and a known volume of bacterial suspension was transferred to each microplate well. Ten microliters of the ethanolic extract (5 mg ml^−1^) of *C. guianensis* leaf was added to the microplate wells and incubated at 35–37°C for 18–20 h. Results were analyzed visually for inhibition zones.

### 2.4. Rats

Healthy inbred male Sprague Dawley rats weighing 180–200 g were used for the study. They were individually housed and maintained on normal food and water *ad libitum*. Animals were periodically weighed before and after the experiment. The rats were anesthetized prior to and during infliction of the experimental wounds. The surgical interventions were carried out under sterile conditions using ketamine anesthesia (120 mg kg^−1^ body weight). Animals were closely observed for any infection and if they showed signs of infection were separated, excluded from the study and replaced.

### 2.5. Animal Ethical Committee Approval

The study was approved by the ethics committee for animal experimentation (AHC06/07/1) by The Faculty of Medical Sciences, The University of the West Indies, St. Augustine.

### 2.6. Excision Wound Model

The anesthetized rats were inflicted with excision wounds as described by Morton and Malone [12]. The dorsal fur of the animals was shaved with an electric clipper and the area of the wound to be created was outlined on the back of the animals with methylene blue using a circular stainless steel stencil. A full thickness of the excision wound of circular area 250 mm^2^ and 2 mm depth was created along the markings with a surgical blade. The animals were randomly divided into two groups of six each: Group 1 (control) animals were applied with petroleum jelly [13]. Animals of Group 2 (experimental) applied with the leaf extract mixed with petroleum jelly at a dose of 250 mg kg^−1^ daily until complete epithelialization. This was done daily for 9 days with full attention to accuracy of dosing.

The wound contraction rate was assessed by tracing the wound on alternate days using transparency paper and a permanent marker. The wound areas recorded were measured using a graph paper. The point at which the eschar fell off without any residual raw wound was considered epithelialization.

### 2.7. Incision Wound Model

The dorsal fur of the anesthetized animals was shaved with an electric clipper. A longitudinal paravertebral incision, 6 cm in length was made through the skin and cutaneous muscle on the back as described by Ehrlich and Hunt et al. [14]. Surgical sutures were applied to the parted skin at intervals of 1 cm. The wounds were left undressed. The animals were randomly divided into two groups of six each. The test group rats were given leaf extract orally in their drinking water at a dose of 250 mg kg^−1^ daily. The controls were given drinking water. As an average, rat consumes 110 ml of water kg^−1^ day^−1^, 250 mg of leaf extract was dissolved in 100 ml of drinking water. The sutures were removed on the 8th post wound day and the treatment was continued. The skin-breaking strength was measured on the 10th day by the method described by Lee [15].

### 2.8. Determination of Wound Breaking Strength

The anesthetized animal was secured to the table, and a line was drawn on either side of the wound 3 mm away from the suture line. The line on either side of the suture was gripped with a forceps one at each end opposed to each other. One end of the forceps was supported firmly, whereas the other was connected to a freely suspended lightweight measuring jar. Water was slowly added continuously till the wound began to gape. As soon as wound gaping appeared the addition of water was stopped. The volume of water was determined and noted as a measure of breaking strength in grams. An average of three readings was recorded for a given incision wound and the mean reading for the group was taken as an individual value of breaking strength [15].

### 2.9. Dead Space Wound Model

Dead space wounds were inflicted by implanting sterile cotton pellets (5 mg each), one on either side of the groin and axilla on the ventral surface of each rat by the technique of D'Arcy et al. (1960) described by Turner [16]. The animals were divided into two groups (*n* = 6). The test group rats were given leaf extract orally in their drinking water at a dose of 250 mg kg^−1^ daily. The controls were given drinking water. On the 10th post-wounding day, the granulation tissue formed on the implanted cotton pellets was carefully removed under anesthesia. The wet weight of the granulation tissue was noted. These granulation tissues were dried at 60°C for 12 h, weighed, and the dry granulation tissue weight was recorded. To the dried tissue 5 ml 6N HCl was added and kept at 110°C for 24 h. The neutralized acid hydrolysate of the dry tissue was used for the determination of hydroxyproline [17]. Wet granulation tissue preserved in 10% formalin was used for histological studies.

### 2.10. Estimation of Hydroxyproline

Dry granulation tissue from both the control and treated groups was used for the estimation of hydroxyproline. Hydroxyproline present in the neutralized acid hydrolysate was subsequently oxidized by sodium peroxide in the presence of copper sulfate followed by complexing with para-dimethylaminobezaldehyde to develop a pink color and that was measured at 540 nm by spectrophotometer [17].

### 2.11. Statistical Analysis

The means of wound area measurements were analyzed using one-way ANOVA descriptive test. The epithelization period, tensile strength, wet and dry weight and hydroxyproline content of the granulation tissue between the test and control groups were compared using independent *t*-test. Data were analyzed using SPSS (Version 12.0, Chicago, USA) and *P*-value was set <.05 for all analyses.

## 3. Results

### 3.1. Phytochemical Analysis

The phytochemical analysis of the leaf extract by qualitative method showed the presence of alkaloids, essential oils, saponins and tannins and absence of triterpenoids and flavonoids.

### 3.2. Excision Wound Model

**Figure 1:**
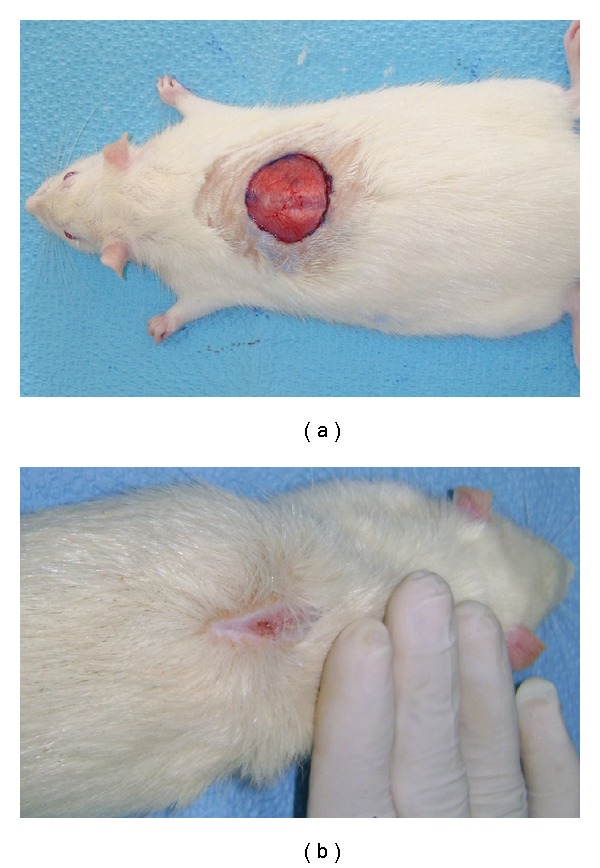
(a) Excision wound on Day 1 (test group animal). (b) Excision wound on Day 15 treated with ethanolic extract of *C. guianensis* leaf (test group animal).

**Figure 2:**
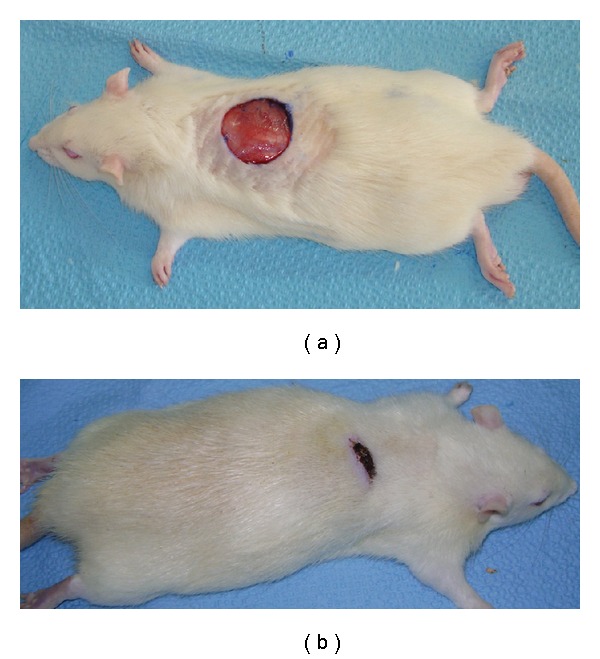
(a) Excision wound on Day 1 (control group animal). (b) Excision wound on Day 15 without any treatment (control group animal).

**Figure 3:**
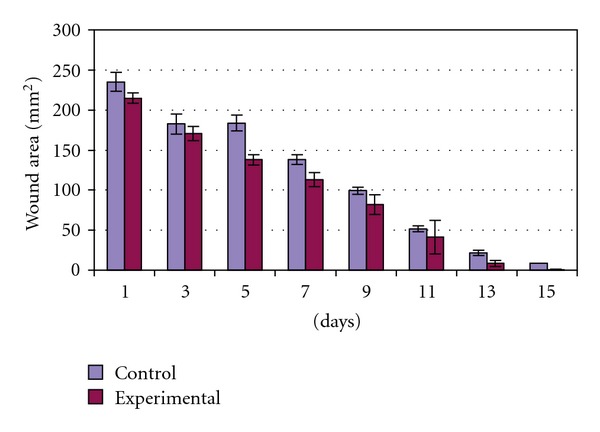
Effect of ethanolic extract of *C. guianensis* leaf on wound contraction in excision wound model.

**Figure 4:**
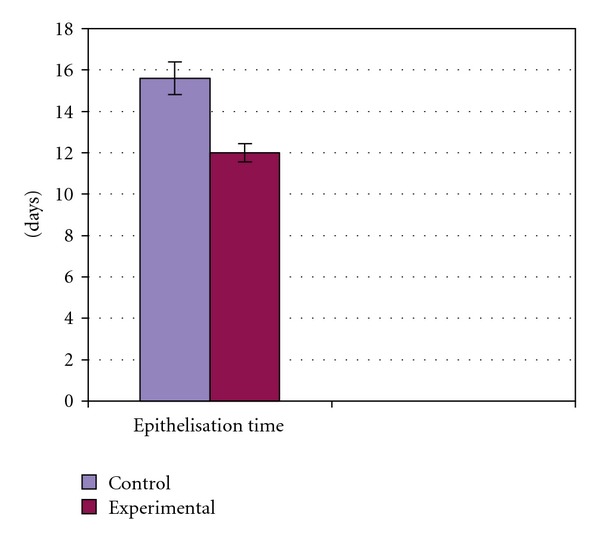
Effect of ethanolic extract of *C. guianensis* leaf on epithelization period.

On Day 15 a significant increase in the wound-healing activity was observed in the animals treated with the *C. guianensis* extract (Figure 1(b)) compared with those who received the placebo control treatments (Figure 2(b)).  Figure 3 shows the effects of the *C. guianensis* leaf extract administered topically on wound healing activity in rats inflicted with the excision wound. In this model *C. guianensis* treated animals were found to epithelize faster (12.00 ± 0.44) when compared with controls (15.60 ± 0.80) (*P* < .03) (Figure 4). The rate of reduction in the wound area of the extract treated animals was 100% when compared with controls (95%) and it was statistically significant (*P* < .03).

### 3.3. Incision Wound Model

**Figure 5:**
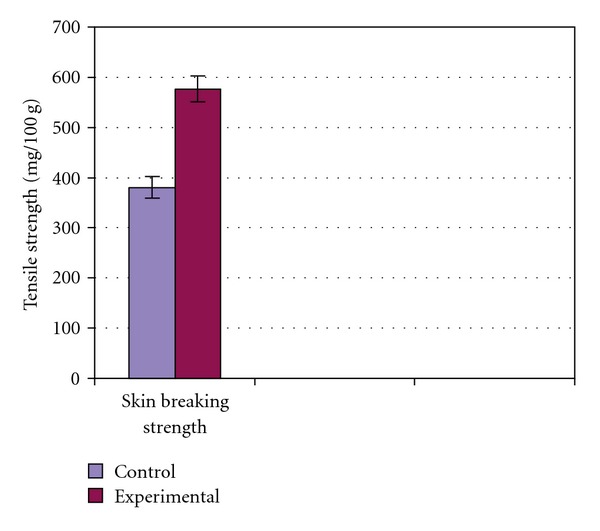
Effect of *C. guianensis* leaf extract on skin breaking strength.

In the incision wound model, a significant increase in the wound breaking strength (576.60 ± 26.03) was observed when compared with the controls (380.10 ± 21.40) (Figure 5).

### 3.4. Dead Space Wound Model

**Figure 6:**
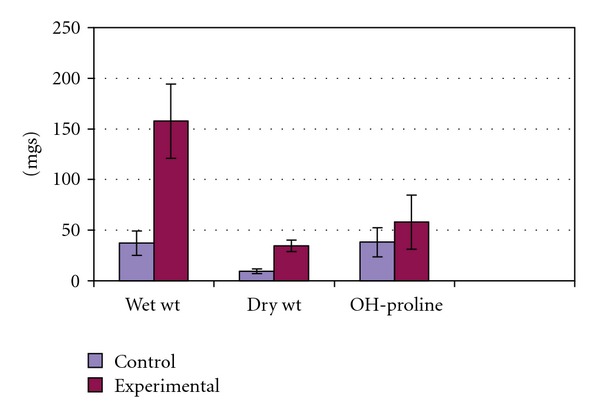
Effect of *C. guianensis* leaf extract on biochemical parameters.

In the dead space wound model, the extract-treated animals showed significantly increased levels of hydroxyproline content (58.00 ± 26.72) as compared with the control group (38.16 ± 14.33) of animals. A significant increase was observed in the wet and dry weight (*P* < .002) of the granulation tissue in the animals treated with the extract (Figure 6).

### 3.5. Antimicrobial Activity

All microbial organisms tested (*P. aeruginosa*, *K. pneumonia*, *E. fecalis*, *E. coli*, *S. aureus* and Methicilin-resistant *S. aureus*) were resistant against *C. guianensis* leaf extract.

## 4. Discussion

Excision, dead space and incision wound models were used to study wound contraction, skin braking strength which are the parameters of tissue cell regeneration, collagenation capacity and mechanical strength of the skin respectively [18, 19].

The ethanolic leaf extract of *C. guianensis* showed increase in the rate of wound contraction, skin breaking strength, the rate of epithelialization, weight and hydroxyproline content of granulation tissue. Granulation tissue formed in the final part of the proliferative phase is primarily composed of fibroblasts, collagen, edema and new small blood vessels. The increased hydroxyproline content of the granulation tissue indicates increased collagen turnover. Collagen, the major component of granulation tissue, strengthens and supports extracellular tissue, which is composed of the amino acid hydroxyproline and it has been used as a biochemical marker for tissue collagen [20].

**Figure 7:**
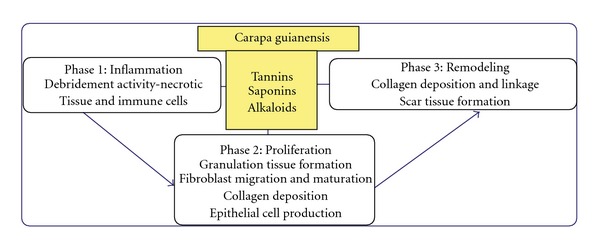
Possible role of phytochemical constituents of *C. guianensis* on wound healing.

Researchers have showed the anti-allergic and anti-inflammatory properties of tetraterpenoids and limnoids isolated respectively from *C. guianensis* [21]. However, our phytochemical analysis of the leaf extract by qualitative analysis showed the presence of alkaloids, saponins, tannins and essential oils and absence of other constituents like triterpenoids and flavonoids. Possibly, constituents like tannins, saponins and alkaloids may play a role in the process of wound healing (Figure 7), however, further phytochemical studies are needed to isolate the active compound(s) responsible for these pharmacological activities.

Several studies demonstrated that the phytochemical constituents present in medicinal plants promote the wound healing process [22–25]. The beneficial response of *C. guianensis* on wound healing may be due to its various reported activities which include antiallergic and antiparasitic [21, 26, 27] effects among its medicinal activities.

While the anti-inflammatory effects of *C. guianensis* are attributed to its possible antihistaminergic activity, the antioxidant activity of *C. guianensis* cannot be discounted. Like honey which is known to have anti-inflammatory, wound-healing promoting action [28] and antibacterial activity [29], *C. guianensis* may also have anti-inflammatory, immunostimulant and pro-healing properties. As *C. guianensis* did not inhibit the growth of microorganisms associated with wound infections, its wound-healing promoting activity is independent of its antimicrobial activity. Future study using isolated active compounds from *C. guianensis* is required to know the reason for negative role of leaf extract in inhibiting the growth of microorganisms.

## 5. Conclusion

We have shown that the leaf extract of *C. guianensis* facilitates wound healing in the experimental animal model. Studies to isolate the active ingredients of the extract that promote wound healing are recommended before proposing its potential application for therapeutic use.
